# A Higher Frequency of CD14^+^CD169^+^ Monocytes/Macrophages in Patients with Colorectal Cancer

**DOI:** 10.1371/journal.pone.0141817

**Published:** 2015-10-28

**Authors:** Chenguang Li, Xiaofan Luo, Yuyang Lin, Xiuqi Tang, Limian Ling, Lei Wang, Yanfang Jiang

**Affiliations:** 1 Department of Colorectal & Anal Surgery, The First Hospital of Jilin University, Changchun, China; 2 Genetic Diagnosis Center, The First Hospital of Jilin University, Changchun, China; 3 Key Laboratory of Zoonosis Research, Ministry of Education, The First Hospital of Jilin University, Changchun, China; Sapporo Medical University, JAPAN

## Abstract

**Objective:**

Monocytes and macrophages can infiltrate into tumor microenvironment and regulate the progression of tumors. This study aimed at determining the frequency of different subsets of circulating monocytes and tumor infiltrating macrophages (TIMs) in patients with colorectal cancer (CRC).

**Methods:**

The frequency of different subsets of circulating monocytes was characterized in 46 CRC patients and 22 healthy controls (HC) by flow cytometry. The frequency of different subsets of macrophages was analyzed in TIMs from 30 tumor tissues and in lamina propria mononuclear cells (LPMCs) from 12 non-tumor tissues. The concentrations of plasma cytokines and carcinoembryonic antigen (CEA) were determined. The potential association of these measures with the values of clinical parameters was analyzed.

**Results:**

In comparison with that in the HC, the percentages of circulating CD14^+^CD169^+^, CD14^+^CD169^+^CD163^+^ and CD14^+^CD169^+^CD206^+^ monocytes and TIMs CD14^+^CD169^+^ as well as IL-10^+^CD14^+^CD169^+^, but not IL-12^+^ CD14^+^CD169^+^ macrophages were significantly increased, accompanied by higher levels of plasma IL-10 in the CRC patients. The percentages of CD14^+^CD169^+^ circulating monocytes and TIM macrophages were associated with the stage of disease and correlated positively with the levels of plasma IL-10 and CEA in CRC patients.

**Conclusion:**

Our data suggest that an increase in the frequency of CD14^+^CD169^+^ cells may be associated with the development and progression of CRC and is concomitant rise of both, pro-tumor (M2-like, IL-10 producing) and anti-tumor (M1-like, IL-12 producing) monocytes and infiltrating macrophages. The frequency of CD14^+^CD169^+^ circulating monocytes and infiltrating macrophages may serve as a biomarker for evaluating the pathogenic degrees of CRC.

## Introduction

Colorectal cancer (CRC) is one of the most prevalent malignant tumors with a high mortality. Its incidence is increasing in many countries and CRC affects > 1.2 million people annually in the world [1]. Previous studies have shown that the development and progression of CRC are associated with local inflammation [2–5]. Indeed, different types of inflammatory infiltrates, particularly for macrophages, are present in the CRC tissues and the balance of protumor and antitumor inflammatory infiltrates is thought to be critical for the development, progression and invasion of CRC [3–4, 6]. Hence, illustration of different types of inflammatory infiltrates and their potential functions will be of great significance in understanding the pathogenesis of CRC.

Macrophages, as professional antigen-presenting cells (APCs) in the innate immune system, are the most abundant immune cell population in the tumor microenvironment [7, 8], and play crucial roles in regulating tissue homeostasis and immune status. Macrophages in the gut tissues can be from their self-renewal [9] and can also stem from the migration of monocytes to the intestinal lamina propria, maintaining gut homeostasis [10, 11]. Macrophages can be classically activated into M1 cells that express inducible nitric oxidase (iNOS), IL-12 and high levels of co-stimulator molecules while resident tissue macrophages and tumor-associated macrophages are likely to express CD163, 206, arginase and IL-10, a M2 phenotype [12–17]. Furthermore, macrophages, like their parental monocytes, express CD14, a co-receptor of TLR4, which has been commonly used for identifying macrophages and monocytes from intestinal mucosa and peripheral blood [18, 19]. Monocytes and tumor infiltrating macrophages (TIM) also express CD169, sialoadhesin, a cell adhesion molecule [20] and MAC387, a marker for immature macrophages [21]. CD169^+^ macrophages are important players in the initiation and progression of inflammatory and autoimmune diseases [22]. Furthermore, CD169^+^ macrophages and monocytes have been thought to dominate pro-inflammation immunity [22–25]. Previous studies have shown that M2 cells in the tumor environment are associated with the progression and metastasis of cancer [15, 26–27]. In addition, CD169^+^ macrophages in regional lymph nodes are associated with a favorable prognosis in CRC patients [28]. However, what type of macrophages in the tumor environment is associated with the progression of CRC remains controversial. It has been confirmed that CD169^+^ macrophages exit in the colon lamina propria, mainly surrounding the crypts and their development is supported by vitamin A in mice [29]. The frequency of circulating CD169^+^ monocytes and tumor infiltrating macrophages in the CRC and their potential association with the progression of CRC have not been clarified.

In the current study, we characterized the frequency of different subsets of CD14^+^CD169^+^ cells in circulating monocytes and in TIMs by flow cytometry and detected the levels of plasma IL-10 and IL-12 as well as carcinoembryonic antigen (CEA) in CRC patients. Furthermore, we analyzed the potential association of the percentages of circulating CD14^+^CD169^+^ monocytes and TIMs with the levels of cytokines and CEA as well as the clinical parameters in CRC patients. We found significantly increased percentages of CD14^+^CD169^+^ in both circulating monocytes and TIMs of CRC patients. In addition, the percentages of CD14^+^CD169^+^ circulating monocytes and TIMs were associated positively with the pathogenic stages of CRC and correlated with the levels of plasma IL-10 and CEA in CRC patients. Thus, our data support the notion that an increased in the frequency of CD14^+^CD169^+^ cells may be associated with the development and progression of CRC and is concomitant rise of both pro-tumor (M2-like, IL-10 producing) as well as anti-tumor (M1-like, IL-12 producing) monocytes and tumor infiltrating macrophages.

## Materials and Methods

### Subjects and samples

The written informed consent was obtained from individual participants. The experimental protocol was established, according to the guidelines of the Declaration of Helsinki and was approved by the Human Ethics Committee of Jilin University (Jilin University, Changchun, China). A total of 46 patients with CRC were recruited at the inpatient service of the Department of Colorectal & Anal Surgery, the First Hospital, Jilin University from February 2013 to December 2014. Individual patients were diagnosed by positive fecal occult blood test, histological examination on biopsied tumor tissues obtained during colonoscopy, and a computed tomography (CT) scan. Individual tumor samples were evaluated for their tumor classification, histological grades, and lymph node metastasis status by pathologists in a blinded manner and staged, according to the tumor-node-metastasis (TNM) classification system of International Union against Cancer (Edition 7) [30]. For stratification analysis, patients with stage I/II of CRC were considered at early stage while those with stage III/IV of CRC were considered at advanced stage. Individual patients were excluded if she/he received radiotherapy, chemotherapy, or immunotherapy. In addition, patients were also excluded if she/he had poor physical condition. Another 22 age-and gender-matched healthy subjects and 12 non-tumor subjects were recruited at the Physical Examination Center and another department of the same hospital. All participants had no history of cancer, autoimmune diseases, recent infectious diseases, inflammatory bowel diseases and familial polyposis. Blood samples were obtained from 46 new onset CRC patients, and 22 healthy controls (HC). Surgical colorectal resection tissue samples were obtained from 30 CRC patients and 12 non-tumor controls, who underwent surgical resection of mixed hemorrhoids. Inflammation may be associated with the development of hemorrhoids. However, a previous study has identified that hemorrhoids appears not to be associated with an increased risk for anal cancer [31] and the nature of inflammation between the environments of CRC and hemorrhoids is different. It is reasonable to use these tissues as the non-tumor controls in our study. The demographic and clinical data of individual participants were collected from hospital records and reviewed by experienced surgeons. Their demographic and clinical characteristics are shown in Table 1.

**Table 1:** The baseline demographic and clinical characteristics of subjects.

Characteristics	Healthy controls	Non-tumor subjects	Early CRC		Advanced CRC	
	blood (n = 22)	tissues (n = 12)	blood (n = 21)	tissues (n = 14)	blood (n = 25)	tissues (n = 16)
**Age (year)**	58(44–69)	57 (46–72)	62 (46–70)	59 (46–71)	61 (43–70)	61 (45–70)
**Sex (Male/female)**	13/9	7/5	14/7	8/6	15/10	10/6
**Tumor location (Colon/Rectum)**	N/A	N/A	6/15	6/8	8/17	5/11
**TNM stage (I/II and III/IV)**	N/A	N/A	6/15 (I/II)	3/11 (I/II)	16/9 (III/IV)	12/4 (III/IV)
**Differentiation (good/moderate/poor)**	N/A	N/A	6/8/7	3/8/3	4/15/6	4/9/3
**Plasma CEA (ng/ml)**	0.52 (0–1.38)	0.23 (0.13–4.21)	3.75 (0.14–33.8)	4.63(0.26–34.2)	24.53(3.75–68.32)*	22.17(2.7–84.3)*
**WBC (×10** ^**9**^ **/L)**	6.43 (4.53–8.27)	6.24 (4.62–9.13)	6.73 (4.21–8.81)	7.23(5.33–9.57)	7.33 (3.73–7.38)	7.35 (4.53–9.31)
**Monocytes (×10** ^**9**^ **/L)**	0.37(0.13–0.51)	0.47 (0.13–0.58)	0.42 (0.23–0.57)	0.53 (0.18–0.62)	0.45 (0.18–0.54)	0.52 (0.16–0.59)

The normal range of CEA: 0–5 ng/ml; white blood cells (WBC): 4–10 ×10^9^/L; monocytes: 0.1–0.6 ×10^9^/L. Quantitative variables were reported as median (range). N/A means not applicable.

*p<0.05 vs. the controls or early patients

### Isolation of peripheral blood mononuclear cells (PBMCs)

Fasting venous blood samples were collected from individual participants, and one portion of blood was used for preparing peripheral blood mononuclear cells (PBMCs) by density-gradient centrifugation using Ficoll-Paque Plus (GE Healthcare). The remaining blood samples were centrifuged for preparing plasma samples.

### Isolation of lamina propria mononuclear cells (LPMCs) and TIMs

The lamina propria mononuclear cells (LPMCs) were isolated from those 12 non-tumor patients and TIMs were isolated from 30 freshly resected surgical tumor specimens, as described previously with minor modification [29, 32]. Briefly, the freshly resected mucosa or CRC tissues were processed within 30 minutes after collection and incubated in calcium and magnesium-free Hanks’ balanced salt solution (HBSS, Sigma-Aldrich) containing 2.5% heat-inactivated fetal bovine serum (FBS) and 1 mM dithiothreitol (Sigma-Aldrich) on ice. The tissues were incubated in calcium and magnesium-free HBSS buffer containing 5 mM EDTA, 1 mM dithiothreitol, 2.5% heat-inactivated FBS, 0.1% v/v β-mercaptoethanol (Sigma-Aldrich, St. Louis, USA) at 37° for 25 min under constant stirring to remove mucus and epithelial cells. Subsequently, the remaining segments were cut into small pieces and digested with 250 μg/ml DNAse (Roche) and 125 μg/ml liberaseTM (Roche) in HBSS at 37° for 30 min under constant stirring. After centrifugation, the cells were re-suspended with RPMI-1640 and filtered through a 40 μM cell strainer, followed by loading on the surface of Ficoll-Paque Plus (GE Healthcare). After centrifugation at 800 g for 20 min, the LPMCs and TIMs were recovered from the interface and washed with PBS twice for flow cytometry.

### Flow cytometry analysis

To determine the frequency of different subsets of CD14^+^ cells, PBMCs or LPMCs (10^6^ cells/tube) were stained with Alexa Fluor 647-anti-CD169 (Biolegend), APC-H7-anti-CD14 (BD PharMingen), BV421-anti-CD163 (BD PharMingen) and PerCP-Cy5.5-anti-CD206 (Biolegend) at 4°C for 30 min respectively. Subsequently, the cells were fixed, and permeabilized using the permeabilization solution (BD Biosciences), followed by intracellular staining with FITC-conjugated anti-MAC387 (Abcam).

Lipopolysaccharide (LPS) can activate monocytes or macrophages by interacting with TLR4 and CD14 and induce the generation of cytokines such as IL-6, IL-10 and IL-12 [33–34]. To detect the function, PBMCs or LPMCs (10^6^ cells/well) were stimulated in duplicate with 50 ng/ml of lipopolysaccharide (LPS) and phorbol myristate acetate (PMA) and 1.0 μg/ml of ionomycin (Sigma-Aldrich) in 10% FBS RPMI-1640 medium at 37°C for 2 hours in 5% CO_2_ and exposed to Brefeldin A (GolgiPlug; BD Biosciences) for another 4 hours, as described previously in our laboratory (21). After being washed, were stained with Alexa Fluor 647-anti-CD169, APC-H7-anti-CD14, fixed, and permeabilized using the permeabilization solution, followed by intracellular staining with PE-CF594-anti-IL-10 (JES3-9D7, BD Biosciences) or PE-anti-IL-12 (BD PharMingen). The matched mouse isotype controls of APC-H7-IgG1, FITC-IgG1, PE-Ig2a, PerCP-Cy5.5-IgG1, PE-CF594-IgG1 and Alexafluor-IgG1 served as the controls. The mononuclear cells were gated according to cell size and internal structure. To better distinguish positive from negative population, we performed Fluorescence Minus One (FMO), by which, the cells were incubated with all of the fluorochromes except for the one that was being measured (CD14, CD169, CD163). The frequency of different subsets of CD14^+^ mononuclear cells was determined by flow cytometry analysis on a BD FACSCalibur (Becton Dickinson) and the data were analyzed using FlowJo 7.6.2 software.

### Enzyme-linked immunosorbent assay (ELISA)

The concentrations of plasma IL-10 and IL-12p70 in individual participants were determined by ELISA using human IL-10 and IL-12p70 ELISA kits, according to the manufacturers’ instruction (R&D Systems). Briefly, human plasma were tested in triplicate and the concentrations of IL-10 and IL-12p70 in individual samples were calculated, according to the standard curves established using recombinant cytokines provided.

### Carcinoembryonic antigen (CEA) assay

The concentrations of plasma CEA in individual samples were determined using an ADVIA Centaur XP immunoassay system (Siemens Healthcare Diagnostics, Tarrytown, USA).

### Statistical analysis

All data are expressed as individual values, median, and range of each group of subjects. The difference between the groups was analyzed using the Mann-Whitney U test. The difference between the tumor and non-tumor tissues was analyzed using the Wilcoxon signed rank test. The potential correlation between variables was analyzed by the Spearman rank correlation test. All statistical tests were performed using SPSS 19.0. A two-sided P value of <0.05 was considered statistically significant.

## Results

### Characterization of circulating CD14^+^CD169^+^ monocytes in CRC patients

To determine the potential roles of circulating CD14^+^CD169^+^ monocytes in the development of CRC, a group of CRC patients and age and gender-matched HC were recruited. As shown in Table 1, there was no significant difference in the distribution of age and gender, the numbers of WBC and monocytes between the patients and HC. Furthermore, there was no significant difference in the tumor location and their differential grades between patients with early and advanced CRC. As expected, the patients displayed significantly higher levels of plasma CEA than the HC (P<0.05).

**Fig 1:**
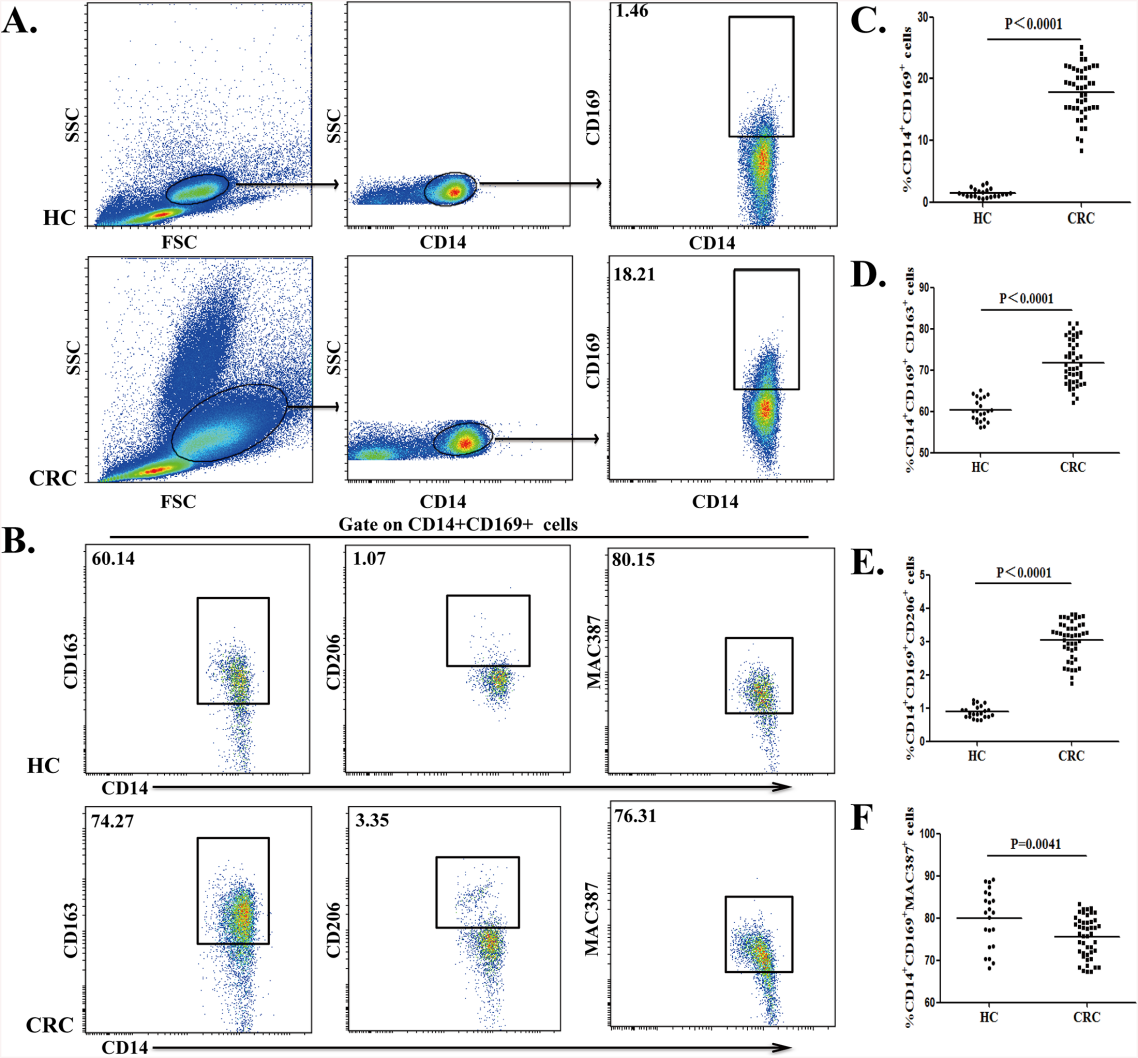
Characterization of circulating CD14^+^CD169^+^ monocytes in CRC patients. Peripheral blood mononuclear cells (PBMCs) were obtained from individual subjects and stained with anti-CD14 and anti-CD169. The frequency of circulating CD14^+^CD169^+^ monocytes was characterized by flow cytometry. The cells were gated initially on mononuclear cells and then on CD14^+^ cells. Subsequently, the percentages of CD14^+^CD169^+^ monocytes were determined. To characterize different subsets of CD14^+^CD169^+^ monocytes, PBMCs were stained with fluorescent antibodies against CD14, CD169 and CD163, CD206 or MAC387. The cells were gated on CD14^+^CD169^+^ monocytes and the percentages of CD14^+^CD169^+^CD163^+^, CD14^+^CD169^+^CD206^+^ and CD14^+^CD169^+^MAC387^+^ macrophages were determined. Data are representative charts of flow cytometry and expressed as the values of individual subjects. The horizontal lines indicate the median for individual groups. (A) Flow cytometry analysis of CD14^+^CD169^+^ monocytes/macrophages. (B) Flow cytometry analysis of different subsets of circulating CD14^+^CD169^+^ monocytes/macrophages. (C) The percentages of CD14^+^CD169^+^ monocytes. (D) The frequency of CD14^+^CD169^+^CD163^+^ macrophages. (E) The frequency of CD14^+^CD169^+^CD206^+^ macrophages. (F) The frequency of CD14^+^CD169^+^MAC387^+^ macrophages.

Previous study have shown that CD14^+^ monocytes represent 80–90% of total circulating monocytes [35]. The CD14^+^, CD169^+^ and CD163^+^ cells were gated according to corresponding isotype controls and FMO controls (S1 Fig). We analyzed the frequency of different subsets of circulating CD14^+^CD169^+^ monocytes from CRC patients and HC by flow cytometry (Fig 1A and 1B). Quantitative analysis revealed that the percentages of circulating CD14^+^CD169^+^ monocytes from the patients were significantly higher than from HC (18.21% vs. 1.42%, P<0.0001; Fig 1C). Hence, significantly increased percentages of circulating CD14^+^CD169^+^ monocytes existed in CRC patients.

Further characterization indicated that the percentage of CD14^+^CD169^+^CD163^+^ cells in total circulating CD14^+^CD169^+^ monocytes from CRC patients were significantly higher than that in HC (74.27% vs. 60.14%, P<0.0001, Fig 1D). Furthermore, the frequency of circulating CD14^+^CD169^+^CD206^+^ monocytes in total circulating CD14^+^CD169^+^ monocytes from CRC patients was also significantly higher than that in HC (3.35% vs. 0.82%, P<0.0001; Fig 1E). In contrast, the percentages of circulating CD14^+^CD169^+^MAC387^+^ monocytes in total circulating CD14^+^CD169^+^ monocytes in the CRC patients were lower than that in HC (76.31% vs. 80.15%, P = 0.0041, Fig 1F). Collectively, CRC patients displayed an imbalance of different subsets of circulating CD14^+^CD169^+^ monocytes.

### Analysis of CD14^+^CD169^+^ macrophages in colorectal neoplasms

**Fig 2:**
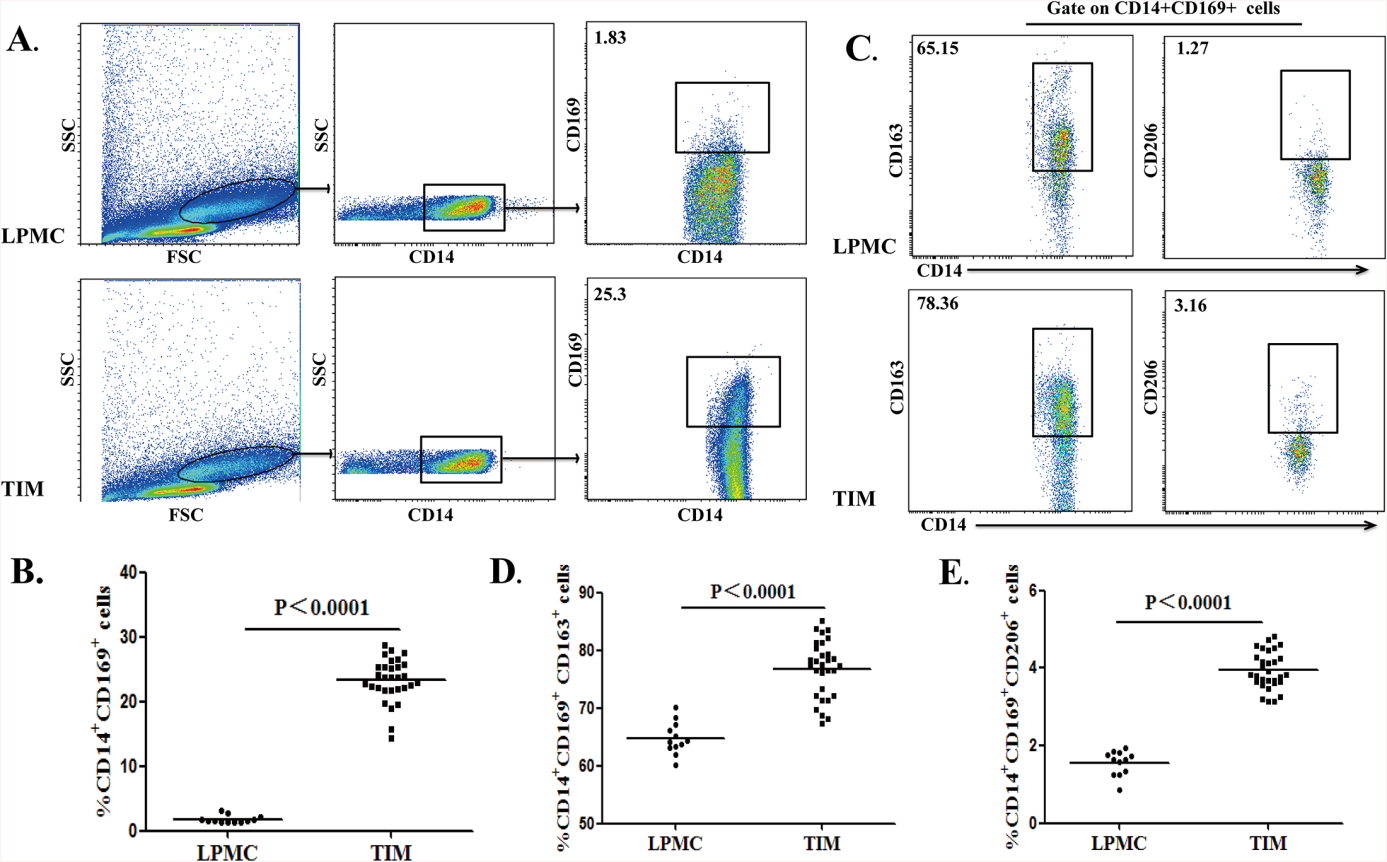
Characterization of CD14^+^CD169^+^ macrophages in the colorectal tissues. A total of 30 fresh surgical CRC tumor tissues were digested for characterization of tumor infiltrating macrophages (TIMs). In addition, 12 lamina propria tissues from non-tumor patients were digested for characterization of macrophages in total lamina propria mononuclear cells (LPMCs). Subsequently, the cells were stained with anti-CD14, CD169 and CD163 or CD206. The frequency of CD14^+^CD169^+^, CD14^+^CD169^+^CD163^+^ and CD14^+^CD169^+^CD206^+^ macrophages in LPMCs and TIMs was determined by flow cytometry. Data are representative charts or expressed as the values of individual subjects. The horizontal lines indicate the median for individual groups. (A) Flow cytometry analysis of CD14^+^CD169^+^ in LPMCs and TIMs. (B) The percentages of CD14^+^CD169^+^ macrophages in LPMCs and TIMs. (C) Flow cytometry analysis of CD14^+^CD169^+^CD163^+^ and CD14^+^CD169^+^CD206^+^ macrophages in total CD14^+^CD169^+^ LPMCs and TIMs macrophages. (D) The frequency of CD14^+^CD169^+^CD163^+^ macrophages in LPMCs and TIMs. (E) The frequency of CD14^+^CD169^+^CD206^+^ macrophages in LPMCs and TIMs.

Peripheral monocytes migrate into organs and tissues to regulate inflammation. To understand the importance of different subsets of CD14^+^CD169^+^ macrophages in the development of CRC, the percentages of different subsets of CD14^+^CD169^+^ macrophages in LPMCs from 12 non-tumor patients and in TIMs from 30 CRC patients were analyzed by flow cytometry (Fig 2A). Although previous studies have shown that macrophages in the gut lamina propria can express different levels of CD14 [18, 36], a fraction of CD14^+^ macrophages were presented in the lamina propria (S1 Fig A) and we focused on this population of intestinal macrophages. The CD14^+^, CD169^+^ and CD163^+^ cells in LPMCs or TIMs were gated according to FMO controls in S1 Fig. The percentages of CD14^+^CD169^+^ macrophages in TIMs were significantly higher than that in LPMCs of non-tumor patients (25.36% vs. 1.83%, P<0.0001; Fig 2B). Furthermore, we analyzed the percentages of CD14^+^CD169^+^CD163^+^ or CD14^+^CD169^+^CD206^+^ cells in LPMCs or TIMs by flow cytometry (Fig 2C). The percentages of CD14^+^CD169^+^CD163^+^ or CD14^+^CD169^+^CD206^+^ macrophages in TIMs were significantly higher than that in LPMCs (78.05% vs. 64.21%, P<0.0001; 3.85% vs. 1.63%, P<0.0001; Fig 2D and 2E). Interestingly, we also detected significantly higher percentages of CD14^+^CD169^+^CD163^+^ or CD14^+^CD169^+^CD206^+^ macrophages in TIMs than in circulating monocytes of CRC patients (data not shown), suggesting that peripheral blood CD14^+^CD169^+^ monocytes may migrate into the lamina propria and became M2-like cells.

### A higher frequency of IL-10^+^CD14^+^CD169^+^ monocytes and macrophages in CRC patients

**Fig 3:**
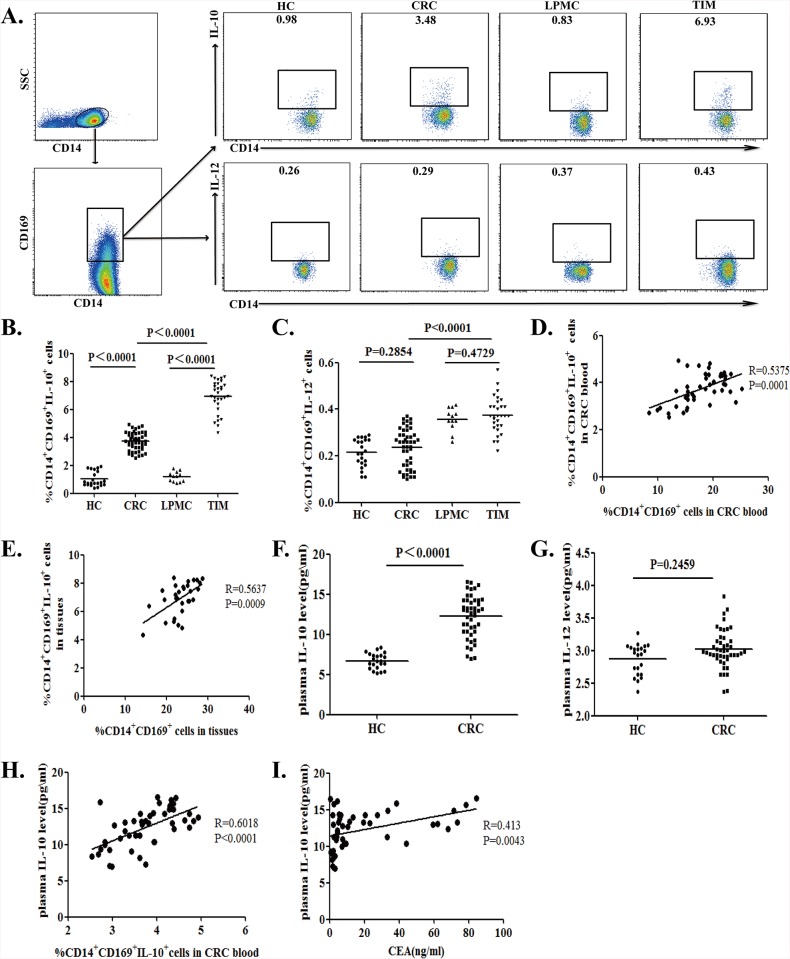
Characterization of CD14^+^CD169^+^IL-10^+^ M2 cells in CRC patients. PBMCs and TIMs were isolated from individual subjects and stimulated with LPS and PMA/ionomycin. The cells were stained with anti-CD169 and anti-CD14 for 30 min, fixed, and permeabilized, followed by intracellular staining with anti-IL-10 or anti-IL-12. The cells were first gated on CD14^+^CD169^+^ cells and the percentages of CD14^+^CD169^+^IL-12^+^ M1 and CD14^+^CD169^+^IL-10^+^ M2 cells in PBMCs or TIMs from individual subjects were determined by flow cytometry. The levels of plasma IL-10 and IL-12 in individual subjects were determined by ELISA. Data are representative charts or expressed as the values of individual patients. The horizontal lines indicate the median for individual groups. (A) Flow cytometry analysis. (B) The percentages of CD14^+^CD169^+^IL-10^+^ M2 cells. (C) The percentages of CD14^+^CD169^+^IL-12^+^ M1 cells. The horizontal lines indicate the median values. (D) The potential association between the percentages of circulating CD14^+^CD169^+^IL-10^+^ cells and CD14^+^CD169^+^ cells in CRC patients. (E) The potential association between the percentages of TIMs CD14^+^CD169^+^IL-10^+^ cells and CD14^+^CD169^+^cells in the CRC tissues. (F) The levels of plasma IL-10. (G) The levels of plasma IL-12. (H) The correlation of plasma IL-10 levels with the percentages of CD14^+^CD169^+^ IL-10^+^ monocytes in PBMCs from CRC patients. (I) The correlation between the levels of plasma IL-10 and CEA in CRC patients.

Previous studies have shown that IL-10^+^ M2 cells are associated with the development of CRC [26, 37–38]. To determine the function of circulating CD14^+^CD169^+^ monocytes and TIMs, the isolated cells were stimulated in vitro and the percentages of IL-10^+^ or IL-12^+^ CD14^+^CD169^+^ circulating monocytes and TIMs were determined by flow cytometry (Fig 3A). The percentages of IL-10^+^CD14^+^CD169^+^ circulating monocytes and TIMs in total CD14^+^CD169^+^ cells from CRC patients were significantly higher than that from the non-tumor subjects (3.79% vs. 0.76%, P<0.0001 for circulating monocytes; 7.12% vs. 1.23%, P<0.0001 for TIMs. Fig 3B). In contrast, there was no significant difference in the frequency of IL-12^+^CD14^+^CD169^+^ circulating monocytes and TIMs between the CRC patients and non-tumor subjects (0.24% vs. 0.22%, P = 0.2854 for circulating monocytes; 0.37% vs. 0.36%, P = 0.4729 for TIMs. Fig 3C). Further analysis of CD14^+^CD169^-^ cells revealed there is no significant difference in the frequency of IL-10^+^ or IL-12^+^CD14^+^CD169^-^ cells in total CD14^+^CD169^-^ cells between the CRC patients and non-tumor subjects in this population (IL-10: 0.017% vs. 0.024%, P = 0.6428 for circulating monocytes; 0.018% vs. 0.014%, P = 0.8125 for TIMs; IL-12: 0.019% vs. 0.013%, P = 0.1628 for circulating monocytes; 0.015% vs. 0.013%, P = 0.8678 for TIMs. S2 Fig). More importantly the percentages of CD14^+^CD169^+^IL-10^+^cells were correlated positively with the percentages of CD14^+^CD169^+^ circulating monocytes and TIMs in the CRC patients (R = 0.5375, P = 0.0001 for circulating monocytes; R = 0.5637, P = 0.0009 for TIMs, Fig 3D and 3E). Hence, CRC patients displayed a higher frequency of IL-10^+^CD14^+^CD169^+^ macrophages.

We measured the concentrations of plasma IL-10 and IL-12 in individual subjects by ELISA. As shown in Fig 3F and 3G, the concentrations of plasma IL-10, but not IL-12, in the CRC patients were significantly higher than that in the HC (P<0.0001). The concentrations of plasma IL-10 were correlated positively with the percentages of circulating IL-10^+^CD14^+^CD169^+^ monocytes (R = 0.6018, P<0.0001; Fig 3H) and the levels of plasma CEA in the CRC patients (R = 0.413, P = 0.0043; Fig 3I).

### Stratification analyses of the frequency of CD14^+^CD169^+^ monocytes and macrophages in CRC patients

**Fig 4:**
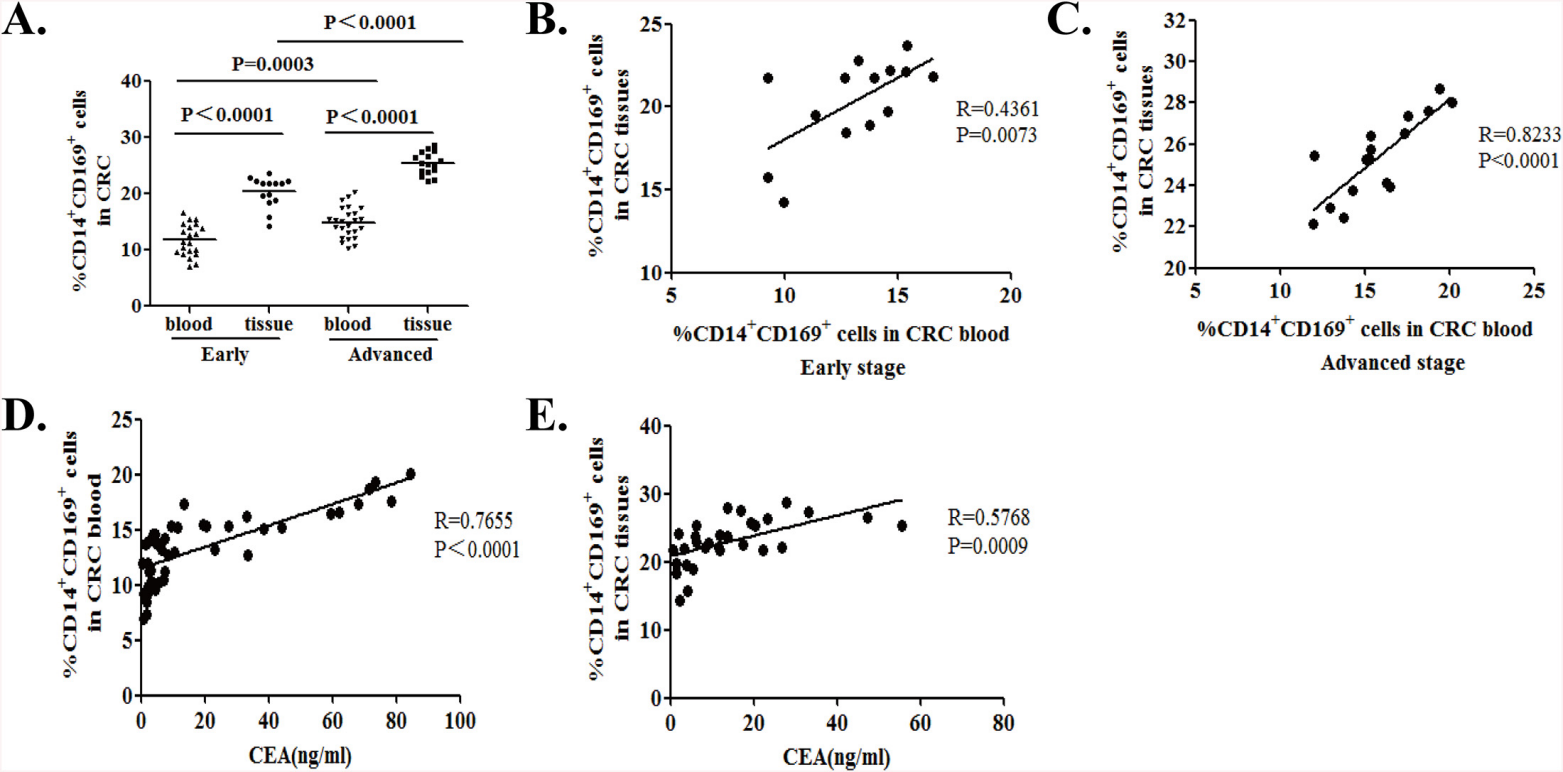
Stratification analysis of the percentages of CD14^+^CD169^+^ circulating monocytes and tumor infiltrating macrophages in CRC patients. The CRC patients were stratified as early (I/II, n = 21) or advanced stage (III/IV, n = 25) and the percentages of CD14^+^CD169^+^ circulating monocytes and TIMs in individual patients were analyzed. Furthermore, the potential association between the percentages of CD14^+^CD169^+^ circulating monocytes or TIMs and the levels of plasma CEA in individual groups of patients were analyzed. Data are the mean of individual subjects. (A) Percentages of CD14^+^CD169^+^ monocytes in PBMCs (n = 21 for early stage, n = 25 for advanced stage of CRC patients) and the percentages of CD14^+^CD169^+^ macrophages in TIMs from early (n = 14) or advanced (n = 16) stage of CRC patients. The horizontal lines indicate the median for individual groups. (B) The correlation between the percentages of CD14^+^CD169^+^ circulating monocytes and TIMs in early stage of CRC patients. (C) The correlation between the percentages of CD14^+^CD169^+^ circulating monocytes and TIMs in advanced stage of CRC patients. (D) The correlation between the levels of plasma CEA and the percentages of CD14^+^CD169^+^ circulating monocytes in the CRC patients. (E) The correlation between the levels of plasma CEA and the percentages of CD14^+^CD169^+^ TIMs in the CRC patients.

Next, we stratified the patients, according to their tumor pathological TNM stages and we found that the percentages of CD14^+^CD169^+^ circulating monocytes were significantly lower in the patients with early stage of CRC than those with advanced stage of CRC (11.81% vs. 15.91%, P = 00003, Fig 4A). Similarly, the percentages of CD14^+^CD169^+^ macrophages in TIMs from the patients with early stage of CRC were significantly lower than that in those with advanced stage of CRC (22.45% vs. 26.93%, P<0.0001, Fig 4A). In addition, the percentages of CD14^+^CD169^+^ circulating monocytes from patients with either early stage or advanced stage of CRC were significantly lower than that in TIMs from the same group of patients (P<0.00001, P<0.0001, respectively; Fig 4A). The percentages of CD14^+^CD169^+^ circulating monocytes were positively correlated with the percentages of CD14^+^CD169^+^ macrophages in TIMs from the patients with early (R = 0.4361, P = 0.0073. Fig 4B) or advanced stage (R = 0.8191, P = 0.0001.Fig 4C) of CRC.

The levels of plasma CEA remain a valuable biomarker for evaluating the CRC progression [39]. Thus, we examined the potential association among the percentages of CD14^+^CD169^+^ circulating monocytes and TIMs with the levels of plasma CEA in the CRC patients. We found that the levels of plasma CEA were correlated positively with the percentages of CD14^+^CD169^+^ circulating monocytes (R = 0.7655, P < 0.0001; Fig 4D) and CD14^+^CD169^+^ macrophages in TIMs (R = 0.5768, P = 0.0009; Fig 4E) in the CRC patients. Together, the percentages of CD14^+^CD169^+^ circulating monocytes and TIMs were positively associated with the pathologic stages of CRC in this population of patients.

## Discussion

To evaluate the marker potential of CD14^+^CD169^+^ cells in the pathogenic progression of CRC, we examined the phenotype and clinical relevance of circulating CD14^+^CD169^+^ monocytes and TIMs in CRC patients. Our data indicated that circulating CD14^+^CD169^+^ M2-like monocytes and TIMs accumulated in the neoplasms and correlated with the levels of plasma IL-10 and CEA in CRC patients. Clearly, the frequency of CD14^+^CD169^+^ circulating monocytes and TIMs were associated with the pathogenic stages of CRC. To the best of our knowledge, this was the first report that significantly higher percentages of circulating CD14^+^CD169^+^ M2-like monocytes or TIMs were associated with pathogenic progression of CRC in humans.

Inflammatory infiltrates in the tumor microenvironment are crucial for the progression and metastasis of tumors [7, 8]. Previous studies have shown that alternatively activated M2 macrophages can participate in the pathogenic process of different types of solid tumors by promoting angiogenesis and metastasis and inhibiting antitumor activity [16, 40–41]. In this study, we detected a significantly higher frequency of circulating and tumor infiltrating CD14^+^CD169^+^CD163^+^ and CD14^+^CD163^+^CD206^+^ M2-like macrophages in CRC patients. These findings suggest that peripheral monocytes may migrate into tumor tissues and regulate tumor growth and immunity. The percentages of CD14^+^CD169^+^ circulating monocytes and TIMs in patients with advanced stage of CRC were significantly higher than those with early stage of CRC. These data further indicated that the frequency of CD14^+^CD169^+^ circulating monocytes and TIMs were associated with the pathogenic degrees of CRC. Our data extended previous observations on inflammatory disease and suggest that CD169^+^ can be expressed by macrophages in tumor tissues [23–25, 42]. Indeed, CD169^+^ macrophages have been detected in normal colon lamina propria [29]. Given that CD169 is an adhesion molecule it is possible that CD14^+^CD169^+^ circulating monocytes may migrate and be activated in the tumor environment to regulate the progression and metastasis of CRC. Our results were different from a previous observation that CD169^+^ macrophages in regional lymph nodes were associated with a favorable prognosis in CRC patients [28]. On the one hand, the difference may stem from different groups of patients with different genetic backgrounds between our study and those of others. On the other hand, CD169 is expressed not only on CD163^+^ or CD206^+^ M2-like macrophages, but also on other subtypes of macrophages. Moreover, tumor-microenvironment consists of many components, which can educate CD169^+^ macrophages to support tumorigenesis. However, further studies are needed to clarify the function of tumor infiltrating CD169^+^ macrophages in the development and progression of CRC.

The levels of plasma CEA have been considered as a biomarker for evaluating the development, progression and recurrence of CRC in the clinic. Previous studies have shown that the frequency of CD169^+^ monocytes is associated with the disease activity in autoimmune diseases, such as systemic sclerosis [23], systemic lupus erythematosus [24] and rheumatoid arthritis [25]. In this study, we detected abnormally higher levels of plasma CEA in CRC patients, particularly in those with advanced stage of CRC. More importantly, the levels of plasma CEA were correlated positively with the percentages of CD14^+^CD169^+^ circulating monocytes and TIMs as well as the levels of plasma IL-10 in CRC patients. Hence, the frequency of CD14^+^CD169^+^ circulating monocytes or TIMs may be valuable for evaluating the pathogenic degrees of CRC. We are interested in further investigating the values of CD14^+^CD169^+^ M2-like cells in evaluating the progression and recurrence of CRC.

We found that the percentages of CD14^+^CD169^+^IL-10^+^ M2-like circulating monocytes and TIMs were significantly higher in CRC patients than in the HC or the non-tumor patients following in vitro activation. However, the percentages of CD14^+^CD169^+^ cells that responded to in vitro stimulation were low although they were similar to 6.3% CD14^+^ monocytes in a previous study [34]. The lower frequency of CD14^+^CD169^+^ cells responding to stimulation may stem from a non-optimal condition for stimulation or low sensitivity to detect cytoplasmic cytokines. In addition, we detected a higher levels of plasma IL-10, but not IL-12 in CRC patients. Previous studies have shown that tumor associated macrophages express a wide range of molecules, such as TGF-β, arginase-1, EGF, VEGF and IL-10 [27, 43–44]. Furthermore, another study has indicated that IL-10^+^ tumor associated macrophages promote the growth of tumors in an IL-10 dependent manner [17]. Indeed, IL-10 secreted by M2-like macrophages induces B2H1 over-expression to inhibit T cells activation and antitumor immunity [45]. The increased frequency of CD14^+^CD169^+^IL-10^+^ macrophages in tumor tissues may create a protumor immunosuppressive microenvironment to promote the progression of CRC. Thus, we are interested in further investigating how the CD14^+^CD169^+^ macrophages in the tumor microenvironment regulate the progression and metastasis of CRC.

We recognized that our study had limitations, including a small sample size and the lack of functional studies of CD14^+^CD169^+^ cells and their interaction with other immunocompetent as well as only centering on CD14^+^ monocytes or macrophages. Therefore, further validation of these findings in a bigger population is warranted to reveal the association between the frequency of CD14^+^CD169^+^ cells and survival of CRC patients.

In summary, our data suggest that an increased in the frequency of CD14^+^CD169^+^ cells may be associated with the development and progression of CRC and is concomitant rise of both pro-tumor (M2-like, IL-10 producing) and anti-tumor (M1-like, IL-12 producing) monocytes and infiltrating macrophages. The frequency of circulating CD14^+^CD169^+^ monocytes or TIMs may serve as biomarkers for evaluating the pathogenic degrees of CRC. To the best of our knowledge, this was the first study to report higher frequency of circulating CD14^+^CD169^+^ monocytes and tumor infiltrating macrophages in patients with newly diagnosed CRC.

## Supporting Information

S1 Fig The gate strategy of CD14^+^, CD169^+^ and CD163^+^ cells in PBMCs and LPMCs. To discriminate positive from negative events for each parameter (CD14, CD169, CD163), the cells were stained with their corresponding isotype controls. Moreover, we also performed FMO to distinguish positive (brown for tissue cells, blue for PBMCs) from negative (pink) populations. (Figure A) The gate strategy of CD14 in LPMCs and PBMCs. (Figure B) The gate strategy of CD169 in LPMCs and PBMCs. (Figure C) The gate strategy of CD163 in LPMCs and PBMCs.
(TIF)

S2 Fig Analysis the frequency of CD14^+^CD169^-^IL-10^+^ or CD14^+^CD169^-^IL-12^+^ cells in PBMCs and LPMCs. To evaluate the intracellular expression of IL-10 and IL-12, the percentages of IL-10^+^ and IL-12^+^CD14^+^CD169^-^ cells were determined by flow cytometry. Data are representative charts or expressed as the values of individual patients. The horizontal lines indicate the median for individual groups. (Figure A) Flow cytometry analysis of CD14^+^CD169^-^IL-10^+^ and CD14^+^CD169^-^IL-12^+^ cells. (Figure B) The percentages of CD14^+^CD169^-^IL-10^+^ M2 cells. (Figure C) The percentages of CD14^+^CD169^-^IL-12^+^ M1 cells.
(TIF)
