# Supplementary figures and images for: A Higher Frequency of CD14+CD169+ Monocytes/Macrophages in Patients with Colorectal Cancer
Source: PLoS One. 2015 Oct 28;10(10):e0141817. doi: 10.1371/journal.pone.0141817 (PMC4625021; doi:10.1371/journal.pone.0141817)

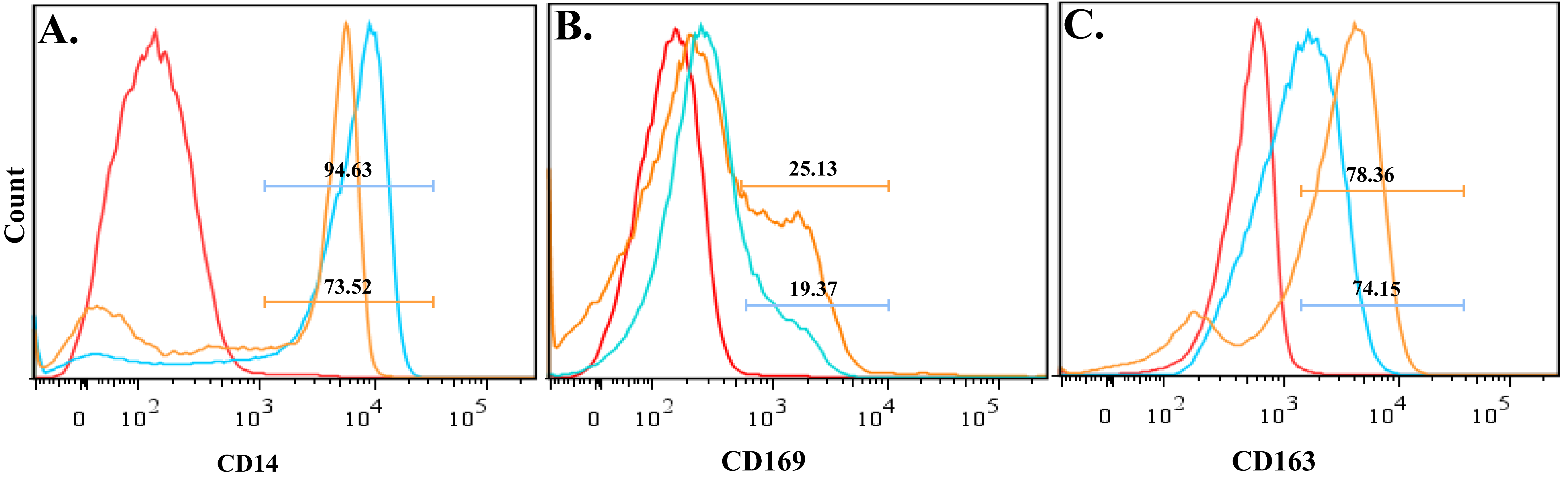

Supplement: S1 Fig — To discriminate positive from negative events for each parameter (CD14, CD169, CD163), the cells were stained with their corresponding isotype controls. Moreover, we also performed FMO to distinguish positive (brown for tissue cells, blue for PBMCs) from negative (pink) populations. (Figure A) The gate strategy of CD14 in LPMCs and PBMCs. (Figure B) The gate strategy of CD169 in LPMCs and PBMCs. (Figure C) The gate strategy of CD163 in LPMCs and PBMCs. (TIF) [file pone.0141817.s001.tif]

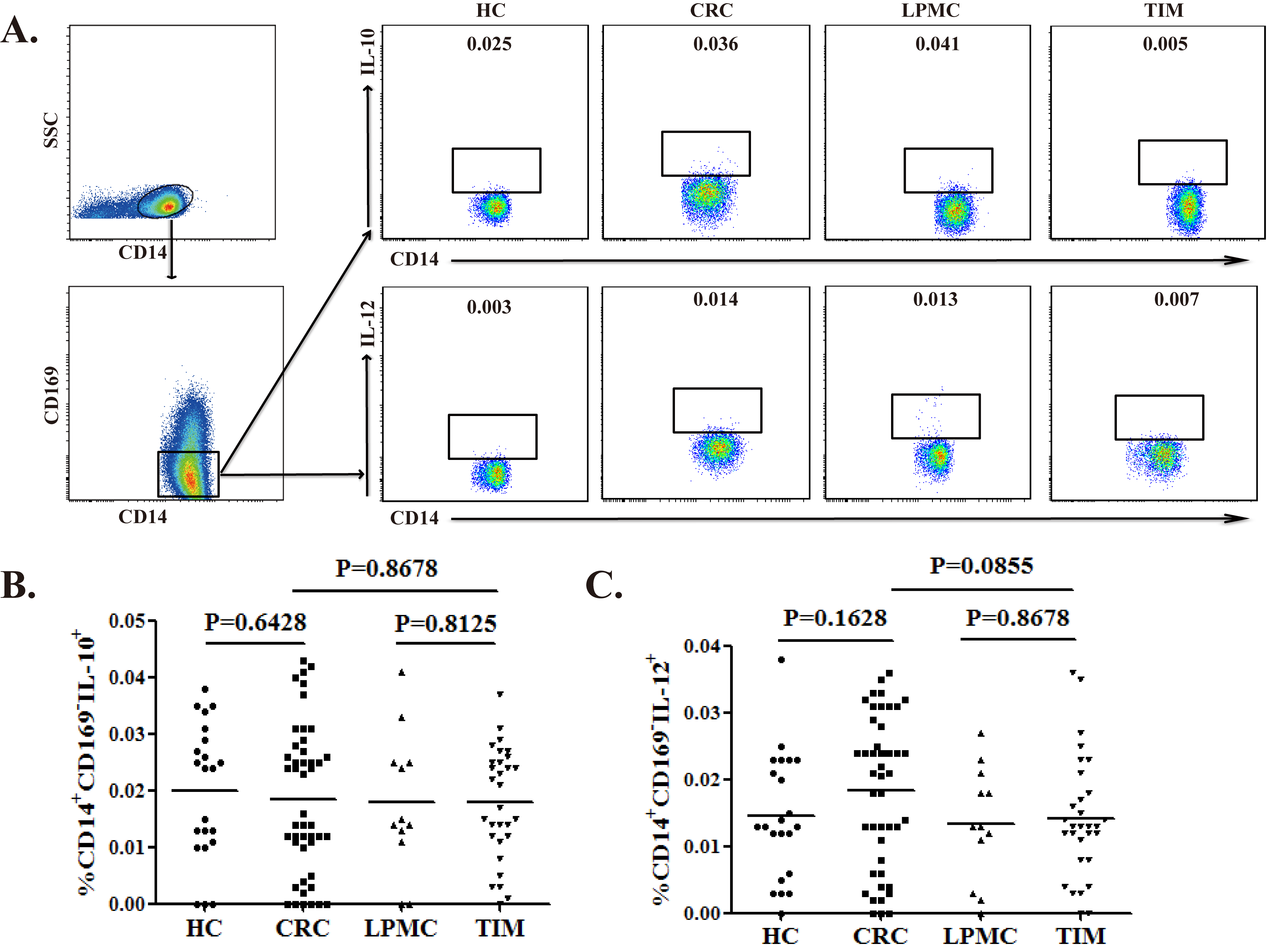

Supplement: S2 Fig — To evaluate the intracellular expression of IL-10 and IL-12, the percentages of IL-10+ and IL-12+CD14+CD169- cells were determined by flow cytometry. Data are representative charts or expressed as the values of individual patients. The horizontal lines indicate the median for individual groups. (Figure A) Flow cytometry analysis of CD14+CD169-IL-10+ and CD14+CD169-IL-12+ cells. (Figure B) The percentages of CD14+CD169-IL-10+ M2 cells. (Figure C) The percentages of CD14+CD169-IL-12+ M1 cells. (TIF) [file pone.0141817.s002.tif]
